# Association of Initial Illness Severity and Outcomes After Cardiac Arrest With Targeted Temperature Management at 36 °C or 33 °C

**DOI:** 10.1001/jamanetworkopen.2020.8215

**Published:** 2020-07-23

**Authors:** Clifton W. Callaway, Patrick J. Coppler, John Faro, Jacob S. Puyana, Pawan Solanki, Cameron Dezfulian, Ankur A. Doshi, Jonathan Elmer, Adam Frisch, Francis X. Guyette, Masashi Okubo, Jon C. Rittenberger, Alexandra Weissman

**Affiliations:** 1 Pittsburgh Post–Cardiac Arrest Service, Department of Emergency Medicine, University of Pittsburgh, Pittsburgh, Pennsylvania

## Abstract

**Importance:**

It is uncertain what the optimal target temperature is for targeted temperature management (TTM) in patients who are comatose following cardiac arrest.

**Objective:**

To examine whether illness severity is associated with changes in the association between target temperature and patient outcome.

**Design, Setting, and Participants:**

This cohort study compared outcomes for 1319 patients who were comatose after cardiac arrest at a single center in Pittsburgh, Pennsylvania, from January 2010 to December 2018. Initial illness severity was based on coma and organ failure scores, presence of severe cerebral edema, and presence of highly malignant electroencephalogram (EEG) after resuscitation.

**Exposure:**

TTM at 36 °C or 33 °C.

**Main Outcomes and Measures:**

Primary outcome was survival to hospital discharge, and secondary outcomes were modified Rankin Scale and cerebral performance category.

**Results:**

Among 1319 patients, 728 (55.2%) had TTM at 33 °C (451 [62.0%] men; median [interquartile range] age, 61 [50-72] years) and 591 (44.8%) had TTM at 36 °C (353 [59.7%] men; median [interquartile range] age, 59 [48-69] years). Overall, 184 of 187 patients (98.4%) with severe cerebral edema died and 234 of 243 patients (96.3%) with highly malignant EEG died regardless of TTM strategy. Comparing TTM at 33 °C with TTM at 36 °C in 911 patients (69.1%) with neither severe cerebral edema nor highly malignant EEG, survival was lower in patients with mild to moderate coma and no shock (risk difference, –13.8%; 95% CI, –24.4% to –3.2%) but higher in patients with mild to moderate coma and cardiopulmonary failure (risk difference, 21.8%; 95% CI, 5.4% to 38.2%) or with severe coma (risk difference, 9.7%; 95% CI, 4.0% to 15.3%). Interactions were similar for functional outcomes. Most deaths (633 of 968 [65.4%]) resulted after withdrawal of life-sustaining therapies.

**Conclusions and Relevance:**

In this study, TTM at 33 °C was associated with better survival than TTM at 36 °C among patients with the most severe post–cardiac arrest illness but without severe cerebral edema or malignant EEG. However, TTM at 36 °C was associated with better survival among patients with mild- to moderate-severity illness.

## Key Points

**Question:**

What is the optimal target temperature for targeted temperature management (TTM) in comatose patients after cardiac arrest?

**Findings:**

In a cohort study of 1319 patients, of whom 911 did not have severe cerebral edema or highly malignant electroencephalogram, TTM at 33 °C was associated with better survival than TTM at 36 °C for patients with the most severe post–cardiac arrest illness, but TTM at 36 °C was associated with better survival in patients with mild- to moderate-severity illness. Patients with severe cerebral edema or highly malignant electroencephalogram had poor outcomes regardless of TTM strategy.

**Meaning:**

The findings of this study suggest that measuring initial illness severity in patients resuscitated from cardiac arrest may guide selection of the optimal TTM strategy.

## Teaser

This cohort study examines whether illness severity is associated with changes in the association between target temperature and patient outcome.

## Introduction

Cardiac arrest and resuscitation often result in brain injury that impairs functional recovery for survivors.^1^ Severe brain injury contributes to in-hospital death for many other patients. Failure to awaken leads to withdrawal of life sustaining therapy (WLST) for most patients with out-of-hospital cardiac arrest who have pulses restored.^2^

Targeted temperature management (TTM) in a mild hypothermic range (32-36 °C) is an intervention that mitigates brain injury in the laboratory.^3^ In clinical trials, treating patients with TTM at 32 to 34 °C resulted in higher survival and better functional recovery than not regulating temperature after out-of-hospital cardiac arrest.^4,5^ However, a trial reported in 2013 did not demonstrate the superiority of TTM at 36 °C vs 33 °C in patients with mild to moderate illness.^6^ Current practice includes TTM for comatose patients after cardiac arrest with a target temperature of 32 to 36 °C.^7,8^ There are few data to guide selection of the target temperature, which relies largely on clinician preference or institutional protocol.^9,10^

Registries reported worse outcomes in cohorts of patients treated after 2013 with TTM at 36 °C compared with patients treated with TTM at 33 °C^10^ or when TTM was not used at all.^11^ A 2019 trial^12^ found TTM at 33 °C to be superior to normothermia in patients with more severe illness. Because the association of TTM at 33 °C with outcomes appears to differ between patients with mild to moderate illness^6^ and severe illness,^12^ we speculated that TTM strategies might have different associations with outcomes among patients with different magnitudes of post–cardiac arrest injury. Therefore, we tested the hypothesis that outcomes differed between patients with TTM at 33 °C and those with TTM at 36 °C, stratified by illness severity.

## Methods

We maintain a quality improvement database of all patients treated for cardiac arrest at our institution. We performed a retrospective cohort study of consecutive patients in this database who were comatose after resuscitation from cardiac arrest and who were treated with TTM. We initiated our study after noting decreased survival in subsets of patients treated with TTM at 36 °C after 2013. The University of Pittsburgh Human Research Protection Office approved retrospective analysis of this database and determined this research to be exempt from the requirement to obtain informed consent. This report follows the Strengthening the Reporting of Observational Studies in Epidemiology (STROBE) reporting guideline for cohort studies.

Our institution is a regional referral center for post–cardiac arrest care.^13,14^ In addition to receiving patients directly from the scene of their cardiac arrest, many patients are initially transported to a local hospital via emergency medical services and are then transferred to our regional center for specialized care. Within our center, a team of physicians provides consultation on most cardiac arrest cases to direct cardiac arrest–specific aspects of care. This group included physicians with prior training in emergency medicine, critical care medicine, or neurology with multiple years of experience as a post–cardiac arrest consultant. Specific domains for which we regiment treatment include TTM, hemodynamic goals, seizure detection and management, ordering and interpretation of testing for neurologic prognostication, referral for rehabilitation, and secondary prevention.^14,15^ Most patients receive electroencephalography (EEG) monitoring during TTM given the incidence of malignant EEG patterns in the post–cardiac arrest population and their association with neurologic outcomes.^16^ Most patients also have routine computed tomography (CT) of the brain within the first hours of admission to assess for early signs of cerebral edema and for neurologic etiologies of cardiac arrest.^17^ Neurologic prognostication uses clinical examination, EEG, evoked potentials, and repeated imaging as ordered and interpreted by our consultation team, according to principles we have previously described.^18^

TTM, initiated as quickly as possible in a mild hypothermia range for 24 hours followed by rewarming at 0.25 °C/h, is routine for all comatose patients (with comatose defined as not following commands), unless there are family or patient directives to limit aggressive critical care. TTM at 33 °C was the routine treatment for all comatose patients after cardiac arrest from 2010 to 2013. From 2014 to 2018, our clinicians selected TTM at 33 °C or 36 °C based on individual preference, anecdotally reporting that their choices were influenced by illness severity.

We use several different body temperature–control devices based on nursing, clinician, and unit preferences. During this period, we used both surface cooling with gel-adhesive pads or water-filled blankets and endovascular cooling devices. Comparisons of devices revealed little difference in performance.^19^ Esophageal temperature was used as the standard measurement site.

Sedation and analgesia with propofol and fentanyl was the most common strategy to suppress shivering, with pharmacologic paralysis as needed. Clinicians used other sedatives, such as midazolam, ketamine, and dexmedetomidine, for individual patients when propofol was not tolerated.

### Exposure

For each patient, we determined whether TTM was targeted at 33 °C or 36 °C. Body temperature goals were explicitly written in the medical record as physician orders. For this analysis, we grouped patients according to the intended regimen. We considered any target of 35 °C or higher part of the TTM at 36 °C group. We considered any target of 34 °C or less part of the TTM at 33 °C group. No included patients had TTM targeted between 34 and 35 °C. We excluded patients for whom TTM strategy could not be determined. We determined actual body temperatures for each patient from the electronic health record. To understand how clinicians selected TTM at 33 °C vs TTM at 36 °C after 2014, 1 of us (C.W.C.) queried each clinician before revealing the scope or findings of this study (eAppendix in the Supplement).

### Primary Outcome

Our primary patient outcome was survival to hospital discharge. Secondary outcomes were survival to hospital discharge without severe functional impairment (modified Rankin Scale [mRS], 0-3) or without neurological devastation (cerebral performance category [CPC], 1-3). An abstractor determines mRS and CPC for all patients with cardiac arrest as part of routine quality assurance from review of the medical record using instruments designed for this purpose.^20^

### Subgroups

We first examined 2 subgroups of patients who rarely survive hospitalization with current medical therapy. First, we examined patients with early, severe cerebral edema on CT of the brain.^16^ We defined severe cerebral edema as a gray-white ratio of radiograph attenuation in Hounsfeld units of less than 1.20 at the level of the basal ganglia. Interrater correlation (>0.64) and test-retest correlation (>0.93) of GWR measurement is high.^17,21^

Second, we examined patients with EEG findings suggestive of irrecoverable primary brain injury. Based on prior literature,^22,23^ we defined *highly malignant EEG* as the absence of any cortical background activity (<2 μV) with intermittent bursts of epileptiform activity, including burst suppression with identical bursts, with or without associated myoclonus. Readers have nearly perfect agreement recognizing this pattern.

Finally, we analyzed patients with neither cerebral edema nor highly malignant EEG, stratified by initial illness severity. We prospectively quantified initial illness severity using the Pittsburgh Cardiac Arrest Category (PCAC) measured during the initial patient evaluation. We derived this 4-level score in cohorts based on the best neurologic examination (based on the motor and brainstem scores of the Full Outline of Unresponsiveness [FOUR] score) and predominant cardiopulmonary failure (hypotension and hypoxemia, based on the cardiovascular and respiratory subscales of the Sequential Organ Failure Assessment [SOFA] score) within 6 hours of restoration of pulses.^24,25^ PCAC is strongly predictive of the probability of survival, multiple organ failure, and awakening, even when calculated by investigators not involved with the patient’s clinical care.^25^

We defined PCAC 1 as awakening and purposeful (FOUR motor, 4); PCAC 2, comatose with preserved brainstem reflexes (FOUR motor and brainstem, 4-7) and without severe cardiopulmonary failure (SOFA cardiovascular and pulmonary, <4); PCAC 3, comatose with preserved brainstem reflexes (FOUR motor and brainstem, 4-7) and severe cardiopulmonary failure (SOFA cardiovascular and pulmonary, ≥4); and PCAC 4, deeply comatose with no movement and missing some brainstem reflexes (FOUR motor and brainstem, <4). When a patient was comatose but the initial neurologic examination was confounded by medications, intoxicants, or chemical paralysis, we defined PCAC as unknown.

### Statistical Analysis

All patients with data on intended TTM therapy were included. We describe continuous and ordinal variables using median and interquartile range (IQR). We describe categorical data using percentages and 95% CIs.

We present the association between illness severity and choice of TTM strategy using odds ratios (ORs) and binary logistic regression. We tested whether clinical variables were associated with the choice of TTM strategy using ORs and binary logistic regression.

We report the relative risk (RR) for survival, awakening, and functional recovery when TTM at 33 °C is selected relative to TTM at 36 °C calculated directly or using log binomial regression.^26^ We used durations and age whenever possible. However, for some models to converge, age was coded as decades or as equal to or older than 70 years, and duration of cardiopulmonary resuscitation (CPR) was coded in 10-minute bins. We believe that 10-minute bins reflect the precision of reported CPR duration when it is not confirmed by automated monitoring.

In the subgroup of patients with neither severe cerebral edema nor highly malignant EEG, we tested for interactions of PCAC with TTM strategy with outcomes. Initially, we included a binary indicator of time period (ie, 2014-2018 vs 2010-2013). These analyses did not reveal any secular trends or interactions with PCAC. Because this indicator was nearly perfectly associated with the use of the TTM at 36 °C strategy, we did not include it in final models. We chose to keep data from 2010 to 2013 because we were certain that comparable patients in that period would have been treated with TTM at 33 °C even if they would have been more likely to receive TTM at 36 °C in 2014 to 2018.

We conducted 2 sensitivity analyses. First, we calculated RRs adjusted for patient and clinical characteristics measured before the TTM exposure that were plausibly associated with outcomes or the choice of TTM, ie, age, sex, in-hospital vs out-of-hospital cardiac arrest location, presence of corneal reflex, presence of pupil reflex, duration of CPR, epinephrine administration and dose, shockable electrocardiogram rhythm, and number of shocks. In final models, we excluded variables with no independent association with outcomes (ie, with *P* > .05). Because epinephrine dose was collinear with duration of CPR and absence of pupil reflex was collinear with PCAC 4, we also dropped these in the final adjusted model.

Second, we created a propensity score for the likelihood to choose TTM at 33 °C vs TTM at 36 °C using the same clinical variables measured before TTM exposure. We then reported the RRs of survival, awakening, and functional recovery with TTM at 33 °C vs TTM at 36 °C in 1:1 propensity matched groups, using calipers of 0.005.

We conducted analyses with Stata version 15.0 (StataCorp). Statistical significance was assessed using 2-sided 95% CIs.

## Results

From 2010 to 2018, we treated 2399 patients after cardiac arrest, of whom 1319 (55.0%) were comatose, eligible for aggressive critical care, and treated with TTM at 36 °C (591 patients [44.8%]; 353 [59.7%] men; median [IQR] age, 59 [48-69] years) or TTM at 33 °C (728 patients [55.2%]; 451 [62.0%] men; median [IQR] age, 61 [50-72] years) (Figure and Table 1). Of the 728 patients who received TTM at 33 °C, 660 (90.7%) achieved a minimum temperature below 34 °C and 178 (24.5%) survived to hospital discharge. Of the 591 patients who received TTM at 36 °C, 173 (29.3%) survived to hospital discharge (Table 1 and Table 2). Most deaths (633 of 968 [65.4%]) resulted after WLST. Body temperatures differed between groups during the first day after cardiac arrest (eFigure in the Supplement).

**Figure.**
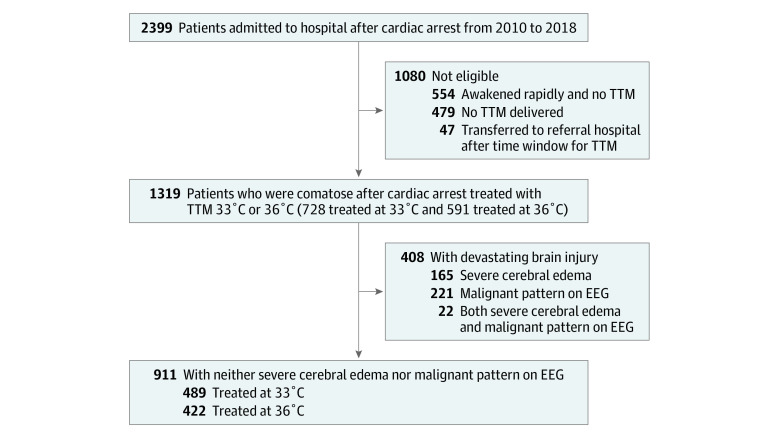
Patients Included in this Study EEG indicates electroencephalogram; TTM, targeted temperature mangagement.

**Table 1.** Characteristics of Patients Treated With TTM at 33 °C or 36 °C

Characteristic	No. (%)	
	TTM 33 °C (n = 728)	TTM 36 °C (n = 591)
Age, median (IQR), y	61 (50-72)	59 (48-69)
Men	451 (62.0)	353 (59.7)
OHCA	622 (85.4)	481 (81.4)
Witnessed collapse	551 (75.7)	467 (79.0)
Initial rhythm		
VF/shockable	195 (26.8)	174 (29.4)
PEA	261 (35.9)	215 (36.4)
Asystole	232 (31.9)	161 (27.2)
Unknown	40 (5.5)	41 (6.9)
Shocked ever	303 (41.6)	261 (44.2)
Shocks, median (IQR), No.	2 (1-4)	2 (1-3)
Received epinephrine	629 (86.4)	510 (86.3)
Epinephrine dose, median (IQR), mg	3 (2-5)	3 (1-4)
Illness severity		
PCAC 2	148 (20.3)	156 (26.4)
PCAC 3	64 (8.8)	87 (14.7)
PCAC 4	466 (64.0)	283 (47.9)
Unable to determine	50 (6.9)	65 (11.0)
Pupil response present	375 (53.9)	338 (59.3)
Corneal response present	181 (30.2)	220 (41.0)
Severe cerebral edema	116 (15.9)	71 (12.0)
Highly malignant EEG	137 (18.8)	106 (17.9)
Year of cardiac arrest		
2010-2013	440 (60.4)	6 (1.0)
2014-2018	288 (39.6)	585 (99.0)
Etiology of arrest		
Acute coronary syndrome	90 (12.4)	88 (14.9)
Coronary angiography	76 (84.4)	70 (79.5)
PCI	72 (80.0)	60 (68.2)
Dysrhythmia	53 (7.3)	49 (8.3)
Structural heart disease	5 (0.7)	4 (0.7)
Systolic heart failure	30 (4.1)	28 (4.7)
Respiratory failure	117 (16.1)	90 (15.2)
Airway obstruction	36 (4.9)	32 (5.4)
Neurological event	4 (0.5)	10 (1.7)
Distributive shock	18 (2.5)	10 (1.7)
Traumatic injury	4 (0.5)	25 (4.2)
Exsanguination	3 (0.4)	16 (2.7)
Toxicological or overdose	96 (13.2)	55 (9.3)
Metabolic derangement	32 (4.4)	18 (3.0)
Other	22 (3.0)	15 (2.5)
Unable to determine	211 (29.0)	146 (24.7)

Abbreviations: EEG, electroencephalogram; IQR, interquartile range; OHCA, out-of-hospital cardiac arrest; PCAC, Pittsburgh Cardiac Arrest Category; PCI, percutaneous coronary intervention; PEA, pulseless electrical activity; TTM, targeted temperature management; VF, ventricular fibrillation.

**Table 2.** Outcomes for Patients Treated With TTM at 33 °C or 36 °C

Outcome	No. (%)	
	TTM at 33 °C (n = 728)	TTM at 36 °C (n = 591)
Survived hospitalization	178 (24.5)	173 (29.3)
ICU length of stay, median (IQR), d	9.5 (6-18)	8 (5-15)
Hospital length of stay, median (IQR), d	17.5 (11-26)	16 (10-25)
Modified Rankin Scale		
0	7 (3.9)	2 (1.2)
1	11 (6.2)	10 (5.8)
2	21 (11.8)	23 (13.3)
3	22 (12.4)	24 (13.9)
4	68 (38.2)	67 (38.7)
5	49 (27.5)	47 (27.2)
Cerebral performance category		
1	18 (10.1)	11 (6.4)
2	25 (14.0)	26 (15.0)
3	115 (64.6)	123 (71.1)
4	20 (11.2)	13 (7.5)
Died in hospital	550 (75.5)	418 (70.7)
ICU length of stay, median (IQR), d	3 (1-4)	2 (1-5)
Mode of death		
Rearrest, intractable shock, or multiple organ failure	121 (22.0)	121 (28.9)
WLST, nonneurologic reasons	50 (9.1)	75 (17.9)
WLST, neurologic prognosis	327 (59.5)	181 (43.3)
Brain death	52 (9.5)	41 (9.8)

Abbreviations: ICU, intensive care unit; IQR, interquartile range; TTM, targeted temperature management; WLST, withdrawal of life-sustaining therapies.

Treating physicians reported that both severe cerebral edema and early status myoclonus were ominous signs that would alter their intensity of critical care, including choice of target temperature (eAppendix in the Supplement). The presence of cerebral edema was associated with the choice of TTM at 33 °C in the entire cohort from 2010 to 2018 (OR, 1.43; 95% CI, 1.03-1.95) but less clearly from 2014 to 2018 (OR, 1.48; 95% CI, 1.00-2.21). Presence of highly malignant EEG was not associated with TTM at 33 °C in the entire cohort from 2010 to 2018 (OR, 1.15; 95% CI, 0.85-1.56) but was associated with TTM at 33 °C from 2014 to 2018 (OR, 1.55; 95% CI, 1.09-2.23).

Severe cerebral edema was present on arrival among 187 patients (14.2%) and highly malignant EEG was present at the beginning of treatment in 243 patients (18.4%) (Figure and Table 1). Overall, 184 patients (98.4%) with severe cerebral edema and 234 patients (96.3%) with highly malignant EEG died, and all survivors had MRS 4 or 5 at hospital discharge. Among subjects with severe cerebral edema, the outcome was most often WLST for neurologic prognosis (93 of 187 [49.7%]), followed by brain death (49 of 187 [26.2%]), cardiovascular collapse or multiple organ failure (36 of 187 [19.3%]), and WLST for nonneurologic reason (6 of 187 [3.2%]). Among patients with highly malignant EEG, the outcome was most often WLST for neurologic prognosis (179 of 243 [73.7%]), followed by WLST for nonneurologic reason (21 of 243 [8.6%]), cardiovascular collapse or multiple organ failure (21 of 243 [8.6%]), and brain death (13 of 243 [5.3%]). Survival did not differ between TTM at 33 °C vs TTM at 36 °C for patients with cerebral edema (2 of 116 [1.7%] vs 1 of 71 [1.4%]) or highly malignant EEG (5 of 137 [3.6%] vs 4 of 106 [3.8%]).

Treating physicians reported that higher illness severity would alter their choice of target temperature (eAppendix in the Supplement). Choice of TTM at 33 °C was associated with PCAC 4 vs PCAC 2 in the entire cohort from 2010 to 2018 (OR, 1.74; 95% CI, 1.33-2.27) and from 2014 to 2018 (OR, 2.42; 95% CI, 1.66-3.53). Among patients with neither severe cerebral edema nor highly malignant EEG, clinician choice of TTM at 33 °C was associated with PCAC 4 vs PCAC 2 from 2010 to 2018 (OR, 1.85; 95% CI, 1.35-2.52) and from 2014 to 2018 (OR, 2.46; 95% CI, 1.57-3.85).

Among 911 patients (69.1%) with neither severe cerebral edema nor highly malignant EEG, we treated 489 (53.7%) with TTM at 33 °C and 422 (46.3%) with TTM at 36° (Table 3). In this cohort, multiple organ failure was the reason for 100 deaths (39.5%) in the TTM at 36 °C cohort and 87 deaths (27.3%) in the TTM at 33 °C cohort; WLST for nonneurologic reasons, 64 deaths (25.3%) and 35 deaths (11.0%), respectively. Survival to hospital discharge differed between different PCAC strata (PCAC 2, 196 of 275 [71.3%]; PCAC 3, 59 of 139 [42.5%]; PCAC 4, 47 of 407 [11.6%]; unknown PCAC, 37 of 90 [41.1%]), and there were robust interactions between PCAC level and effect of TTM at 33 °C (TTM × PCAC 3: RR, 2.00; 95% CI, 1.32-3.04; TTM × PCAC 4: RR, 3.36; 95% CI, 1.59-7.10). Likewise, there were interactions between PCAC level and choice of TTM at 33 °C for mRS (TTM × PCAC 3: RR, 4.89; 95% CI, 1.73-13.8) and CPC outcomes (TTM × PCAC 3: RR, 1.76; 95% CI, 1.14-2.74; TTM × PCAC 4: RR, 3.35; 95% CI, 1.50-7.49).

**Table 3.** Outcomes for Patients With Neither Severe Cerebral Edema nor Highly Malignant EEG Treated With TTM at 33 °C or 36 °C

Outcome	No. (%)	
	TTM at 33 °C (n = 489)	TTM at 36 °C (n = 422)
Survived hospitalization	171 (35.0)	168 (39.8)
ICU length of stay, median (IQR), d	9 (5-18)	8 (5-15)
Hospital length of stay, median (IQR), d	17 (11-26)	16 (10-24)
Modified Rankin Scale		
0	7 (4.1)	2 (1.2)
1	11 (6.4)	10 (6.0)
2	21 (12.3)	23 (13.7)
3	22 (12.9)	24 (14.3)
4	67 (39.2)	67 (39.9)
5	43 (25.1)	42 (25.0)
Cerebral performance category		
1	18 (10.5)	11 (6.5)
2	25 (14.6)	26 (15.5)
3	113 (66.0)	122 (72.6)
4	15 (8.8)	9 (5.4)
Died in hospital	319 (65.2)	253 (60.0)
ICU length of stay, median (IQR), d	3 (1-5)	2 (1-5)
Mode of death		
Rearrest, intractable shock, or multiple organ failure	87 (27.3)	100 (39.5)
WLST, nonneurological reasons	35 (11.0)	64 (25.3)
WLST, neurological prognosis	174 (54.5)	75 (29.6)
Brain death	22 (6.9)	15 (5.9)

Abbreviations: ICU, intensive care unit; IQR, interquartile range; TTM, targeted temperature management; WLST, withdrawal of life-sustaining therapies.

PCAC strata differed in association of TTM choice with outcomes (Table 4). For patients in PCAC 2, TTM at 36 °C was associated with more survival vs TTM at 33 °C (110 of 141 [78.0%] vs 86 of 134 [64.2%]; risk difference for TTM at 33 °C vs 36 °C, −13.8%; 95% CI, −24.4% to −3.2%) and CPC 1 to 3 at hospital discharge (105 [74.5%] vs 82 [61.2%]; risk difference for TTM at 33 °C vs 36 °C, –13.3%; 95% CI, –24.2% to −2.3%). For patients in PCAC 3, TTM at 33 °C vs TTM at 36 °C was associated with more survival (32 of 58 [55.1%] vs 27 of 81 [33.3%]; risk difference, 21.8%; 95% CI, 5.4% to 38.2%) and mRS 0 to 3 at hospital discharge (14 [24.1%] vs 5 [6.2%]; risk difference, 18.0%; 95% CI, 5.8% to 30.2%). For patients in PCAC 4, TTM at 33 °C vs TTM at 36 °C was associated with more survival (39 of 259 [15.1%] vs 8 of 148 [5.4%]; risk difference, 9.7%; 95% CI, 4.0% to 15.3%) and CPC 1 to 3 at hospital discharge (34 [13.1%] vs 7 [4.7%]; risk difference, 8.4%; 95% CI, 3.0% to 13.7%).

**Table 4.** Outcomes Stratified by Initial Illness Severity for Patients With Neither Severe Cerebral Edema nor Highly Malignant EEG Treated With TTM at 33 °C or 36 °C

Illness Severity	No./Total No. (%)		Risk difference with 33 °C, % (95% CI)	RR (95% CI)	Adjusted RR (95% CI)^a^	RR in propensity matched sample (95% CI)^b^
	TTM at 33 °C	TTM at 36 °C				
Survival						
Overall	171/489 (35.0)	168/422 (39.8)	–4.8 (–11.1 to 1.5)	0.88 (0.74 to 1.04)	0.88 (0.76 to 1.01)	0.89 (0.74 to 1.08)
PCAC 2	86/134 (64.2)	110/141 (78.0)	–13.8 (–24.4 to −3.2)	0.82 (0.71 to 0.96)	0.79 (0.68 to 0.93)	0.84 (0.71 to 0.99)
PCAC 3	32/58 (55.1)	27/81 (33.3)	21.8 (5.4 to 38.2)	1.66 (1.13 to 2.43)	1.47 (1.01 to 2.13)	1.50 (0.90 to 2.51)
PCAC 4	39/259 (15.1)	8/148 (5.4)	9.7 (4.0 to 15.3)	2.79 (1.34 to 5.80)	1.89 (0.89 to 4.01)	1.50 (0.63 to 3.54)
No PCAC	14/38 (36.8)	23/52 (44.2)	–7.3 (–27.8 to 13.0)	0.83 (0.50 to 1.40)	0.82 (0.45 to 1.50)	1.00 (0.47 to 2.14)
MRS 0-3 at hospital discharge						
Overall	61/489 (12.5)	59/422 (14.0)	–1.5 (–5.9 to 2.9)	0.89 (0.64 to 1.25)	0.96 (0.70 to 1.34)	0.75 (0.51 to 1.09)
PCAC 2	33/134 (24.6)	44/141 (31.2)	–6.6 (–17.1 to 4.0)	0.79 (0.53 to 1.16)	0.88 (0.60 to 1.28)	0.85 (0.56 to 1.30)
PCAC 3	14/58 (24.1)	5/81 (6.2)	18.0 (5.8 to 30.2)	3.91 (1.49 to 10.3)	2.98 (1.11 to 8.02)	2.67 (0.76 to 9.33)
PCAC 4	10/259 (3.9)	3/148 (2.0)	1.8 (–1.4 to 5.1)	1.90 (0.53 to 6.81)	1.09 (0.29 to 4.06)	0.67 (0.11 to 3.92)
No PCAC	4/38 (10.5)	7/52 (13.4)	–2.9 (–16 to 10.5)	0.78 (0.25 to 2.48)	0.77 (0.22 to 2.74)	1.00 (0.23 to 4.37)
CPC 1-3 at hospital discharge						
Overall	156/489 (31.9)	159/422 (37.8)	–5.8 (–12.0 to 0.4)	0.85 (0.71 to 1.01)	0.85 (0.73 to 0.99)	0.86 (0.70 to 1.05)
PCAC 2	82/134 (61.2)	105/141 (74.5)	–13.3 (–24.2 to −2.3)	0.82 (0.70 to 0.97)	0.80 (0.68 to 0.94)	0.83 (0.70 to 1.00)
PCAC 3	28/58 (48.3)	27/81 (33.3)	14.9 (–1.5 to 31.4)	1.45 (0.96 to 2.17)	1.37 (0.91 to 2.06)	1.36 (0.80 to 2.31)
PCAC 4	34/259 (13.1)	7/148 (4.7)	8.4 (3.0 to 13.7)	2.78 (1.26 to 6.10)	1.76 (0.78 to 3.97)	1.43 (0.56 to 3.63)
No PCAC	12/38 (31.6)	20/52 (38.5)	–6.9 (–26.7 to 12.9)	0.82 (0.46 to 1.46)	0.70 (0.33 to 1.45)	1.00 (0.47 to 2.14)

Abbreviations: CPC, cerebral performance category; MRS, modified Rankin Scale; PCAC, Pittsburgh Cardiac Arrest Category; TTM, targeted temperature management; RR, relative risk.

^a^ Adjusted for age older than 70 years, duration of cardiopulmonary resuscitation, shockable initial rhythm, and absence of pupillary light reflex on initial examination.

^b^ Propensity match includes age, sex, out-of-hospital vs in-hospital location of cardiac arrest, witnessed collapse, shockable initial rhythm, and duration of cardiopulmonary resuscitation. Matched groups include 384 pairs (median bias, 3.2%) overall, 112 pairs (median bias, 4.3%) for PCAC 2, 40 pairs (median bias, 13.0%) for PCAC 3, 126 pairs (median bias, 4.0%) for PCAC 4, and 20 pairs (median bias, 6.6%) for no PCAC.

After adjustment for other variables and in propensity-matched groups, RR estimates were in the same direction but with wider confidence intervals for most outcomes (eg, survival among patients in PCAC 2: crude RR, 0.82; 95% CI, 0.50-1.40; adjusted RR, 0.82; 95% CI, 0.45-1.50; RR in propensity-matched groups, 0.89; 95% CI, 0.74-1.08) (Table 4). The exception was mRS at hospital discharge among patients in PCAC 4, for which estimates differed between crude (RR, 1.90; 95% CI, 0.53-6.81), adjusted (RR, 1.09; 95% CI, 0.29-4.06), and propensity-matched analyses (RR, 0.67; 95% CI, 0.11-3.92).

## Discussion

Choice of TTM at 36 °C vs TTM at 33 °C from 2014 to 2018 was associated with lower survival and functional recovery among patients with the most severe post–cardiac arrest illness, after exclusion of patients with severe cerebral edema and highly malignant EEG patterns. It is perilous to infer a causal connection based on these observational cohort data. However, our observations are consistent with results of a recent clinical trial that randomly assigned patients with nonshockable rhythms to TTM at 33 °C or TTM at 37 °C^12^ and with observational studies noting decreased survival among patients after adoption of a TTM at 36 °C strategy.^10^ Taken together, these data are consistent with a differential effect of TTM strategy based on illness severity.

The beneficial effect of lower body temperatures for more severe illness severity has biological plausibility. Reducing brain temperature can reduce seizure incidence,^27^ cerebral edema,^28^ intracranial pressure,^29^ and metabolic demand during marginal perfusion.^30^ Patients in PCAC 4 are defined by clinical absence of cortical function and have a higher incidence of cerebral edema and malignant EEG patterns. These patients might benefit from neurological treatment, such as hypothermia, that has little incremental value in patients with preserved cortical function (ie, those in PCAC 2). Patients in PCAC 3 are defined by cardiopulmonary failure, which can result in poor tissue perfusion and oxygenation. The brains of these patients might benefit from a reduction in metabolic demand during critical hypoperfusion.

An equally plausible alternative explanation for the current results is clinician bias in selecting TTM strategy. Interviews suggested that treating physicians were more likely to select TTM at 36 °C for patients they believed had nonsurvivable illness or with antecedent goals of care that limited critical care support to minimize delays in implementation of end-of-life care. Although we accounted for the obvious signs of severe cerebral edema and malignant status myoclonus, more subtle features of the patients, especially the values and preferences of surrogate decision-makers, are not captured in the abstracted data. If clinical judgment is accurate, assignment of a few more moribund patients to TTM at 36 °C would bias our results to favor TTM at 33 °C. The higher proportion of deaths in TTM at 36 °C vs TTM at 33 °C attributed to multiple organ failure (39.5% vs 27.3%) or WLST for nonneurologic reasons (25.3% vs 11.0%) suggests that this bias is present (Table 3). However, prediction of expected recovery varies greatly between clinicians and may not be very accurate, especially early in the clinical course.^31^ Regardless of accuracy, clinician bias can influence treatment and family decisions about continued aggressive critical care. Even the PCAC score itself may bias clinicians, although we note that this score correlates with patient outcomes even when calculated asynchronously for patients treated by a separate clinical group in another hospital.^25^

The excellent outcome for patients in PCAC 2, in whom survival consistently exceeded 60%, is reassuring. Patients presenting with favorable clinical signs immediately after restoration of pulse may not require or benefit from a TTM at less than 36 °C, and a less intensive TTM strategy might reduce complications. This fact may explain some of the neutral findings in randomized clinical trials, which excluded patients with the most severe injuries. For example, the TTM trial excluded patients resuscitated from asystole.^6^ The final cohort in that trial had short no-flow times, usually preserved brainstem reflexes, and most closely resembled the patients in PCAC 2 encountered at our center. Survival for the patients in PCAC 2 in this study is comparable with survival for the TTM trial cohort. An ongoing clinical trial will better assess whether rigorous fever control is superior to TTM at 33 °C in this patient phenotype.^32^

Few studies or trials choose therapy based on initial illness severity or even report initial illness severity after cardiac arrest. The present analysis confirms how strongly illness severity is associated with expected survival and outcomes^24,25^ and how illness severity can interact with response to TTM.^34^ A previous study from our center^36^ illustrated how illness severity interacted with outcomes after coronary angiography. In the future, clinicians may select specific therapies based on illness severity. Several measures of illness severity after cardiac arrest are available.^24,25,37^ To advance our understanding of pathophysiology and to find optimal therapies, future studies and randomized clinical trials in patients with cardiac arrest must stratify patients according to severity.

### Limitations

This study has limitations. We emphasize caution using these observational data to guide clinical practice. Observational data are prone to unmeasured biases, and we documented that physicians are consciously using clinical gestalt to select TTM strategies. Adjusted analyses using measured variables can never completely remove this type of bias. However, these data agree with clinical trials^12,33^ and cohort studies^10,35^ suggesting a preference for lower temperatures in patients with more severe post–cardiac arrest illness. Laboratory^3^ and clinical trial data^35^ also support a preference for longer TTM durations in patients with severe illness. At the same time, our data support a preference for TTM at 36 °C for patients with mild to moderate injury. Choice of either TTM therapy seems to be ineffective for patients with severe cerebral edema or highly malignant EEG, including malignant status myoclonus.

## Conclusions

In this study, choosing TTM at 33 °C was associated with better outcomes than TTM at 36 °C for patients with severe post–cardiac arrest illness, but TTM at 36 °C was associated with better survival in mild- to moderate-severity illness. The findings of this study suggest that measuring initial illness severity in patients resuscitated from cardiac arrest may guide selection of the optimal TTM strategy.

## Notes

Supplement. **eAppendix. **Physician Preferences for TTM at 33 ºC versus TTM at 36 ºC **eFigure.** Actual Body Temperature of Patients by Group
