# Supplementary material for: Association of Initial Illness Severity and Outcomes After Cardiac Arrest With Targeted Temperature Management at 36 °C or 33 °C
Source: JAMA Netw Open. 2020 Jul 23;3(7):e208215. doi: 10.1001/jamanetworkopen.2020.8215 (PMC7378753; doi:10.1001/jamanetworkopen.2020.8215)
Supplement: Supplement. — eAppendix. Physician Preferences for TTM at 33 ºC versus TTM at 36 ºC eFigure. Actual Body Temperature of Patients by Group [file jamanetwopen-3-e208215-s001.pdf]

Supplementary Online Content

Callaway CW, Coppler PJ, Faro J, et al. Association of initial illness severity and outcomes after cardiac arrest with targeted temperature management at 36 °C or 33 °C. *JAMA Netw Open*. 2020;3(7):e208215. doi:10.1001/jamanetworkopen.2020.8215

eAppendix. Physician Preferences for TTM at 33 ºC versus TTM at 36 ºC
eFigure. Actual Body Temperature of Patients by Group

This supplementary material has been provided by the authors to give readers additional information about their work.

© 2020 Callaway CW et al. *JAMA Network Open.*

eAppendix. Physician Preferences for TTM at 33 ºC versus TTM at 36 ºC

Comments on why physician would favor TTM 33ºC
PCAC 3 if no contraindication, PCAC 4
PCAC 3 and PCAC 4, unless cerebral edema or status myoclonus
PCAC 3 with less than withdrawal on motor exam
PCAC 2 and 3, plus PCAC 4 if anticipate chance of survival
Young with little room for brain swelling on CT scan
GWR >1.2 but not normal on CT scan
Deeply brain injured but maybe salvageable

Comments on why physician would favor TTM 36ºC
PCAC 2
Cerebral edema or status myoclonus (multiple physicians)
Bleeding or hemodynamic instability
Devastating injury or anticipated brain death (multiple physicians)
Anticipated non-survival (multiple physicians)
PCAC 3 with at least withdrawal on motor exam
GWR 1.1-1.2 on initial CT scan

© 2020 Callaway CW et al. *JAMA Network Open.*

eFigure. Actual Body Temperature of Patients by Group

Median temperature and IQR for subjects treated with TTM at 33ºC or TTM at 36ºC versus time after cardiac arrest. One or more temperatures above 38.5ºC occurred during the first 3 days in 97 (13%) subjects in the TTM 33ºC group versus 111 (19%) subjects in the TTM 36ºC group.

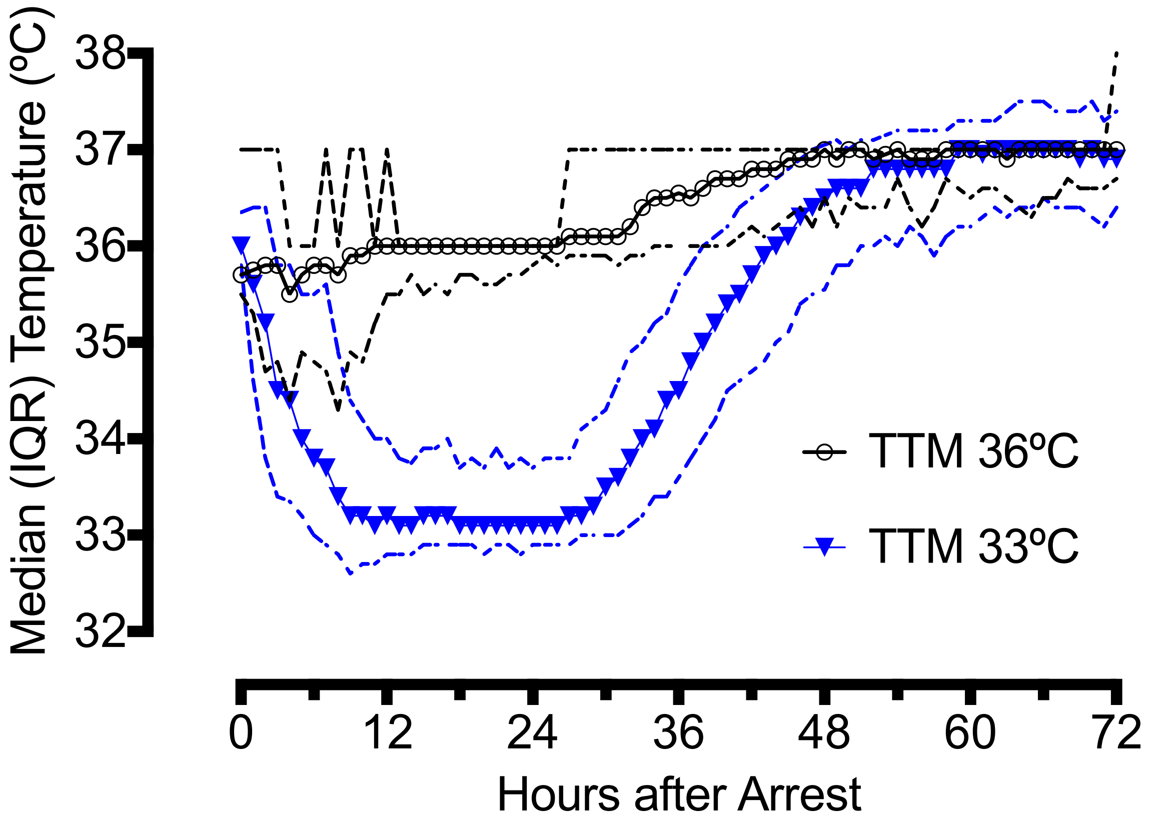

© 2020 Callaway CW et al. *JAMA Network Open.*
